# Pattern recognition of hematological profiles of tumors of the digestive tract: an exploratory study

**DOI:** 10.3389/fmed.2023.1208022

**Published:** 2023-08-16

**Authors:** Miguel A. Santos-Silva, Nuno Sousa, Marina Majar, Miguel Machado, Joana Reis, Joao C. Sousa

**Affiliations:** ^1^Life and Health Sciences Research Institute (ICVS), School of Medicine, University of Minho, Campus de Gualtar, Braga, Portugal; ^2^ICVS/3B's – PT Government Associate Laboratory, Braga/Guimarães, Portugal; ^3^Clinical Academic Center-Braga (2CA), Braga, Portugal; ^4^Association P5 Digital Medical Center (ACMP5), Braga, Portugal; ^5^Clinical Pathology Department, Hospital de Braga, Braga, Portugal

**Keywords:** cancer, pattern recognition, hematological profile, anatomical location, digestive tract tumor

## Abstract

**Aims:**

In this study, we aimed to apply laboratory blood analysis to identify the hematological (based on hemoglobin concentration, erythrocytes, hematocrit, and RDW count) profiles associated with the most prevalent forms of digestive tract malignancies. Furthermore, we aimed to evaluate how these profiles contributed to distinguishing these tumors at diagnosis.

**Methods:**

We collected data from the date of ICD-10 diagnostic coding for C15 esophagus, C16 stomach, C18 colon, and C19 rectum tumors of 184 individuals. The statistical analysis and data visualization approaches, notably the heat map and principal component analysis (PCA), allowed for creating a summary hematological profile and identifying the most associated parameters for each pathologic state. Univariate and multivariate data modeling and ROC analysis were performed in both SPSS and Python.

**Results:**

Our data reveal unique patterns based on tumor development anatomical location, clustering the C18 colon and C19 rectum from the C15 esophagus and C16 stomach. We found a significant difference between C16 stomach carcinoma and the other tumors, which substantially correlated with raised RDW in conjunction with low hemoglobin concentration, erythrocytes, and hematocrit counts. In contrast, C18 colon carcinoma had the higher red blood cell count, allowing for the best classification metrics in the test set of the binary logistic regression (LR) model, accounting for an AUC of 0.77 with 94% sensitivity and 52% specificity.

**Conclusion:**

This study emphasizes the significance of adding hematological patterns in diagnosing these malignancies, which could path further investigations regarding profiling and monitoring at the point of care.

## Introduction

Esophageal, gastric, and colorectal carcinomas are among the most prevalent malignancies and account for a significant portion of cancer-related morbidity and death globally, with a considerable burden on healthcare (1). In fact, the global growth of digestive tract cancers has impacted millions of people. According to the International Agency for Research on Cancer (IARC), colorectal cancer (CRC) has a 6.1% incidence rate and a 9.2% fatality rate, followed by stomach (8.2%), esophagus (5.3%), and rectum (3.2%) (2). Diagnosis of these malignancies is well standardized in current clinical decision support systems, through the use of several technologies, from DNA sequencing, chromosomal and immunology analysis, endoscopy (3), colonoscopy (4), histology, and blood tests, among others (5). Nevertheless, blood tests remain the primary method used by clinicians to assess the hematological profile and conduct subsequent investigations, influencing nearly 70% of the medical decisions (6).

Generally, low levels of RBC, Hb, and HTC and high values of WBC, PLT, and RDW are associated with cancer diagnosis (7).

RDW was previously identified as a biomarker of right-sided CRC cases, with an 84% sensitivity and an 88% specificity (8). It was also discovered to exhibit increased values in patients with esophageal (9) and gastric (10) cancers, with a significant correlation with the digestive tract tumor stage. As a prevalent clinical condition in digestive tract tumors, anemia recognition by measuring Hb over time is also considered an important factor for improving CRC detection and further diagnosis (11).

Artificial intelligence (AI)-driven research has been developing remarkable results in feasibility studies involving routine blood tests with cancer diagnosis (12–14) and prognosis (15, 16). The machine-learning ColonFlag^®^ model developed by Kinar et al. used a decision-trees algorithm to predict the risk of CRC based on age, sex, and cell blood counts (CBC), demonstrating increased sensitivity (AUC = 0.81) than anemia guidelines (AUC = 0.76), especially considering a 6-month period before diagnosis (12). Hornbrook et al. validated the ColonFlag^®^ model in a US-insured population by confirming model applicability in CRC diagnosis (AUC = 0.80), and also highlighting model performance based on the anatomical location of the carcinoma, with better metrics in the cecum and ascending colon rather than in transverse, sigmoid, and rectum (13).

Pattern recognition (PR) refers to the AI's ability to infer underlying patterns (regularities, trends, or anomalies) in the data (17). Different from statistics, PR automatically extracts actionable knowledge from complex datasets. When leveraged by machine- or deep-learning algorithms, trained models could be used to predict similar structures. Nonetheless, the degree of model explainability decreases in deep learning, limiting the model's applicability (18). In blood data, there is a vast amount of non-appraised clinical information that cannot be 100% perceived by clinicians, endorsing the use of PR in processing, patterning, and flagging if necessary (19).

Thus, we thought of interest to go beyond the classic studies evaluating potential biomarkers for diagnosis and explore hematological data from patients with these pathologies from a distinct perspective. Indeed, herein, we assessed whether erythrocytes, hemoglobin, hematocrit, and RDW enable profile distinction between the location of digestive tract tumors and if they can (and contribute to) distinguish them at the time of diagnosis.

## Methods

### Study design and population

This study is a retrospective observational study of Portuguese people diagnosed with the most prevalent oncological diseases at the Hospital de Braga between January 2018 and 2021. The Hospital's oncological registry issues an identification code to combine the patient's clinical information longitudinally. We collected anonymous data from adult patients (18+ years of age) at random prior to the diagnosis codification date, specifically demographics (sex and age), laboratory blood tests (complete blood count and routine biochemistry), and the diagnosis (ICD-10). We gathered information on 184 patients with digestive system cancers, including the esophagus, stomach, colon, and rectum. Laboratory blood tests were manually filtered to identify four distinct parameters conducted in every case, in a total of 760 tests. The study was approved by the Braga Hospital Ethics Committee under the project “Application of machine learning for hematological diagnosis” (Protocol Code 191_2022).

### Data source, measurement, and features

Patient anonymized demographics and laboratory blood test data were retrieved in the Clinic Academic Center (2CA, Braga, Portugal) of the Hospital de Braga. Cell blood count, including erythrocytes, hematocrit, red cell distribution width, and hemoglobin concentration, were analyzed in the clinical pathology laboratory using standard methods (Sysmex XE-2100, Sysmex Inc., Mundelein IL, USA). Disease diagnosis was codified according to the International Statistical Classification of Diseases and Related Health Problems 10th revision (ICD-10). Time before disease codification, that is, timeframe, was converted in a scalar of days to comply with anonymization requirements, and it was calculated as the absolute difference between the date of disease codification (0) with the date of analysis, that is, 731—starting period of the retrospective analysis.

### Statistical analysis

The statistical analysis explored the ICD-10 studied diseases as a function of sex, age, and metabolites. This analysis was performed with SPSS (IBM SPSS Statistics for Mac, version 26, IBM Corp., NY, USA). Continuous variables were evaluated for normal distribution with histograms (skewness and kurtosis) and described using mean and standard deviation (SD). Skewed continuous variables were reported with median and interquartile range (IQR). The timeframe of analysis was selected to zero, corresponding to the codification date. One-way ANOVA (with Tukey's HSD as a *post-hoc* test) and the Kruskal–Wallis test were used to compare groups for parametric and non-parametric variables, respectively. The statistical significance level was set at 0.05.

### Pattern recognition and data modeling

After z-score normalization, PCA and heat map (supervised visualization tool) were used to explore and visualize patterns across the research laboratory blood tests within the study groups. The “Clustvis” online tool, found at https://biit.cs.ut.ee/clustvis/, was used to create the scores plot of the PCA and the heat map, which was computed using the correlation function for “clustering distance”, the “tightest cluster first” for clustering and functionalized with the RdBu palette, from −2 to 2. Principal components (PC1 and PC2) with respective loadings were also made available for interpretation and parameters influence in disease discrimination. Preprocessing was accomplished in Python 3.10.2 (VS 1.64.2) through the exploration of specific libraries for data acquisition (accessing database's raw data), curation (removal of incorrect values or characters and merging of separate intraday analysis), and normalization (i.e., z-score). Univariate and multivariate data modeling and ROC analysis were computed in both SPSS and Python. The classification performance was measured by the area under the receiver operator curve (AUROC), which ranged from zero to one, with one being a perfect classifier. Because our experiments had four classes, the AUROC was calculated as one vs. rest for each class. The F1-score, which ranges from 0 to 1, represents the balanced mean of precision and recall.

## Results

The study cohort included 184 patients (60% men and 40% women, median age of 69 years, IQR 58–81) with complete blood analysis performed on the diagnostic date (t0). C18 colon (*n* = 84) was the most prevalent group, followed by C16 stomach (*n* = 66), C19+C20 rectum (*n* = 19), and C15 esophagus (*n* = 15). On the day of codification, 89, 66, 19, and 16 clinical blood analyses had been completed on each illness, respectively.

Except for age and RDW, which are shown with the median and interquartile range due to their continuously skewed distribution, Table 1 depicts the quantitative features of patients in terms of mean and standard deviation. Indeed, neither age nor RDW was found to have significant differences across groups; however, this assumption was only confirmed for age since the Mann–Whitney tests revealed significant differences in RDW between C15 esophagus and C16 stomach (*p* = 0.035), and between C16 stomach and C18 colon (*p* = 0.026).

**Table 1:** Descriptive statistics of demographics and laboratory blood tests according to each digestive tract tumor.

	**C15 Esophagus**	**C16 Stomach**	**C18 Colon**	**C19+C20 Rectum**	**Sig**.
Number of blood tests	16	66	89	19	
Number of patients	15	66	84	19	
Sex (f/m)	3/12	24/42	40/44	8/11	
Age (years)^*^	68 (57–82)	69 (61–81)	68 (58–83)	71 (53–78)	0.919
Erythrocytes ( × 10^6^/μL)	3.80 ± 0.58	3.73 ± 0.75	4.33 ± 0.81	4.07 ± 0.87	< 0.005
Hemoglobin (g/dL)	11.38 ± 1.79	9.96 ± 2.65	12.05 ± 2.45	11.72 ± 2.43	< 0.005
Hematocrit (%)	34.07 ± 4.94	30.50 ± 7.03	36.39 ± 6.56	35.71 ± 6.92	< 0.005
RDW (%)^*^	13.0 (12.3–15.2)	15.0 (12.8–18.4)	13.7 (12.5–15.2)	14.1 (13.1–14.6)	0.056

^*^The normal distribution was not confirmed.

Non-parametric tests (Kruskal–Wallis test) were used to compare groups, and results are provided as the median and interquartile range (IQR).

Considering the remaining hematological parameters, statistically significant differences in erythrocytes, hemoglobin, and hematocrit were found, with superior levels in C18 colon, followed by C19 rectum, C15 esophagus, and C16 stomach. *Post-hoc* analysis using Tukey's HSD revealed further significant differences in erythrocytes between C16 stomach and C19 rectum (*p* = 0.001), as well as statistically significant variances in hemoglobin between C16 stomach and C18 colon (*p* < 0.001), and C19 rectum (*p* = 0.034), and also in hematocrit between C16 stomach and C18 colon (*p* < 0.001), and C19 rectum (*p* = 0.016).

**Figure 1:**
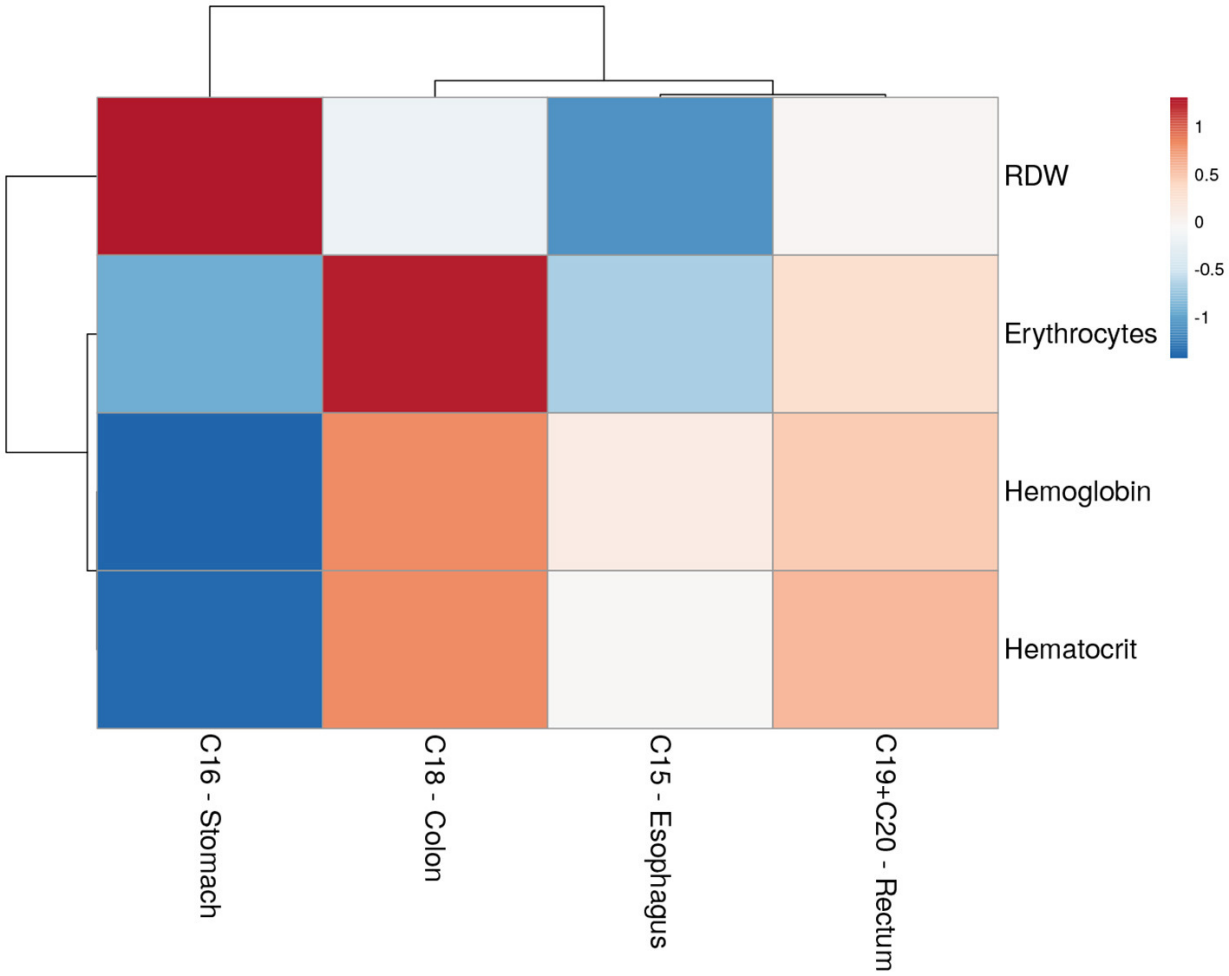
Correlation heat map between each blood parameter and the tumors of the digestive tract.

The heat map allows for the visualization of laboratory blood test patterns (rows) in relation to the researched disease categories (columns) (Figure 1). The matrix profiles each disease location based on its correlation with each metabolite, with a strong positive correlation shown in red and a significant negative correlation shown in blue. Moreover, the map groups diseases and metabolites by nodding (branching), approaching C15 esophagus and C19 rectum to C18 colon, and separating C16 stomach from the preceding. Indeed, C16 stomach displays the strongest correlated profile, with high values of RDW and low hematocrit, hemoglobin, and erythrocytes levels. C18 colon displays a strong correlation between high levels of erythrocytes and moderate association with hemoglobin and hematocrit, with a practically negligible association with RDW. Low RDW levels are closely associated with C15 esophagus, which is also characterized by low erythrocyte count. The C19 rectum displays the weakest correlation profile.

**Figure 2:**
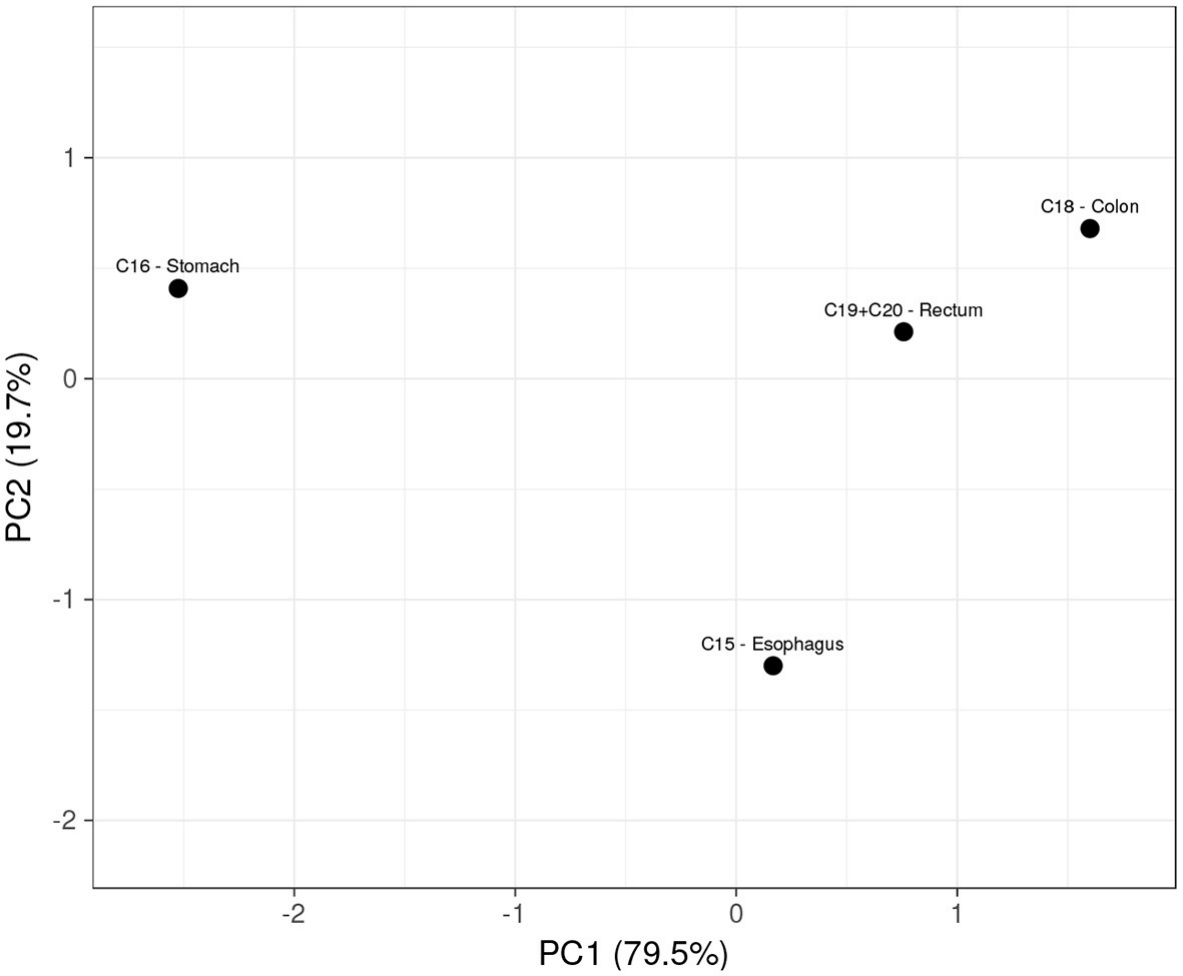
Scores plot of the PCA showing a relationship between digestive tract tumors, based on the four hematologic parameters.

Considering the previously exhibited correlations, each laboratory blood test was subjected to a ROC analysis to assess its predictive ability among the studied groups. We calculated the confidence intervals at a 95% confidence level for the associated AUC values of each parameter. Only RDW in C16 stomach (0.52–0.70), erythrocytes (0.62–0.77), hemoglobin (0.59–0.74), and hematocrit (0.60–0.76) in C18 colon revealed potential diagnostic value (AUC > 0.5) in distinguishing the respective tumor groups. The studied parameters displayed a lower ability to distinguish C15 esophagus or C19 rectum from the others. Both sensitivity and specificity maximized by the Youden index were computed for the parameters with diagnostic ability: While RDW in C16 stomach achieved 52 and 73%, C18 colon was predictable by erythrocytes with 60 and 71%, hemoglobin with 76 and 52%, and by hematocrit with 80 and 51%, respectively. Principal component analysis is a dimensionality reduction algorithm that was used to maximize the variance between disease groups through the linear merging of the blood parameters and extract information regarding the latent variables (principal components) that explain the distribution of the scores. The first principal component of the PCA plot (PC1, Figure 2) separates C16 stomach from the other tumors, with a direct influence of the higher values of RDW (−0.40) together with the low levels of hemoglobin (0.56), hematocrit (0.56), and erythrocytes (0.47). The second principal component, which explains the remaining 20% of the PCA, is strongly determined by erythrocytes (−0.61) and RDW (−0.79), wherein C15 esophagus is clustered from the remaining tumors due to the low levels of RDW and erythrocytes. A small overlap between C19 rectum and C18 colon was also verified, which should not be sufficient for the separation of tumors.

**Figure 3:**
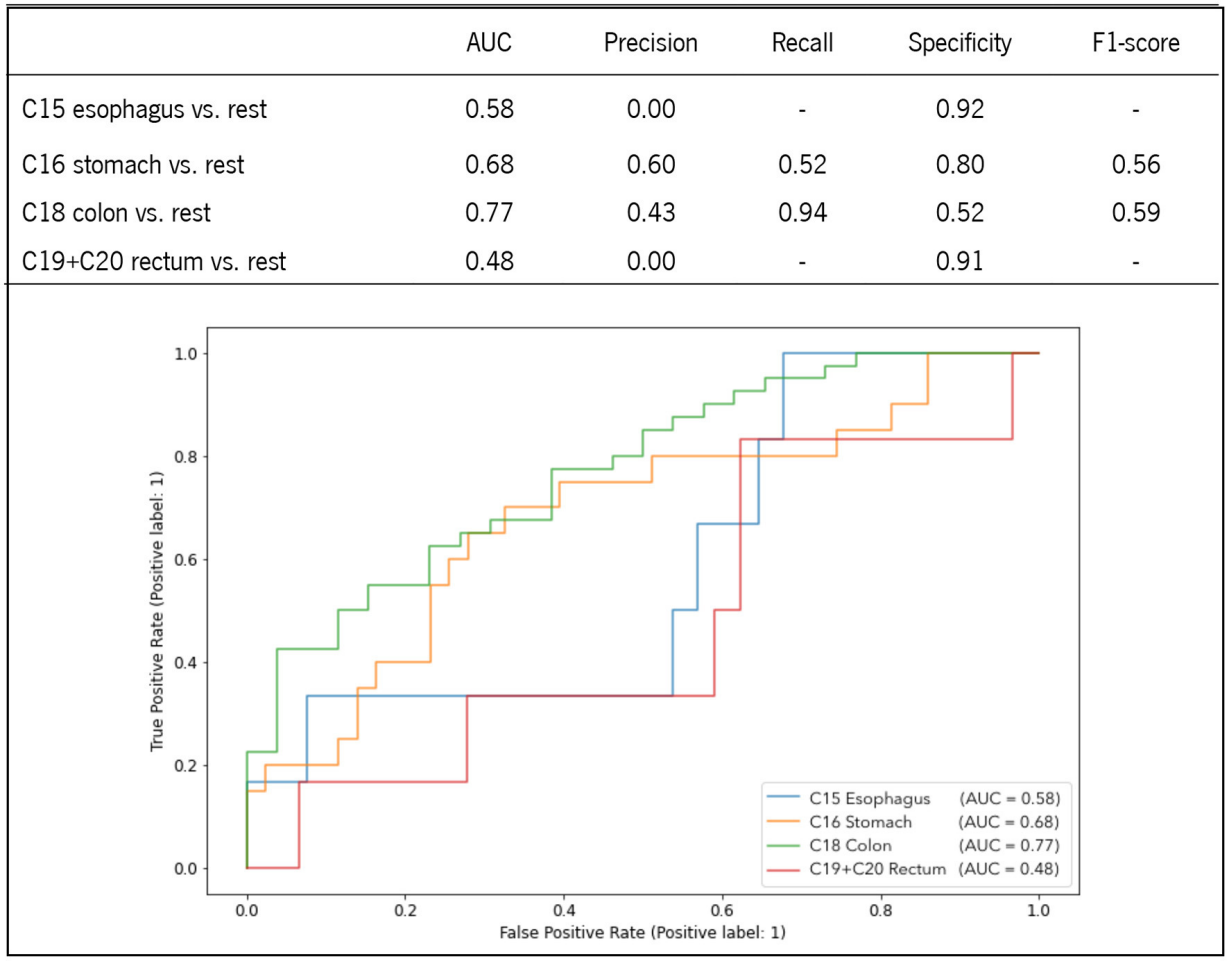
ROC curves and performance metrics of the LR prediction models for the classification of each digestive tract tumor.

LR was computed to evaluate the predictive ability of the studied hematological parameters for the classification of each digestive tract tumor. To preserve representative distribution between training and test sets, data were split in a 70:30 ratio using stratified group k-fold, ensuring similar proportions in class distribution for each subset. Figure 3 highlights the predictive performance of each class by using data from the test set. As expected, the aggregation of the studied metabolites enabled higher performance metrics when compared to univariate analysis. Both C18 colon and C16 stomach exhibited superior performance, confirming the findings of the heat map, which indicated higher correlation profiles for these tumors. Specifically, C18 colon achieved the highest AUC of 77% with an excellent sensitivity (94%) and moderate specificity (52%). C16 stomach showed to be more precise (60%), with moderate recall (52%) and higher specificity (80%). Still, the f1-score of each tumor is comparable. C15 esophagus and C19+C20 rectum displayed lower performance metrics due to the lack of positive samples. Indeed, the confusion matrix of the predicted test set failed to demonstrate sensitivity and f1-score due to the absence of true positive predictions. C15 esophagus and C19+C20 rectum were less correlated with the studied blood parameters, and their AUC was 58 and 48%, respectively.

## Discussion

In this study, we aimed to analyze whether routine hematological parameters were able to perform profile distinction between the location of digestive tract tumors and whether they could contribute to distinguishing them at the time of diagnosis. We applied a cross-decomposition algorithm (PCA) to maximize the variance among the studied 184 blood tests and identify the latent variables that contributed to the model distribution.

Solely using the combination of the hemoglobin concentration, erythrocytes, hematocrit, and RDW count, the model clustered C16 stomach from the other tumors (C15 esophagus, C16 stomach, C18 colon, and C19 rectum) in a linear merge between high RDW count with low hemoglobin, erythrocytes, and hematocrit levels (PC1), explaining nearly 80% of the variance. The remaining 20% belonged to the second principal component, which distinguished the C15 esophagus from the others due to its low erythrocytes and RDW count. Interestingly, no significant overlap was found between C18 colon and C19 rectum in the scores plot (Figure 2, first quadrant), neither across the hematological parameters compared in the statistical analysis of the two groups (Table 1), which is consistent with previous studies indicating similar patterns (of miRNA in the case) between C18 colon and C19 rectum due to the common hindgut region of tumor development (20). Moreover, C15 esophagus and C16 stomach were clustered from the colorectal malignancies in the second (–PC1, +PC2) and fourth (+PC1, –PC2) quadrants of the PCA, keeping the different regions of tumor development separated.

The heat map analysis allowed an easier visualization of the hematological patterns and provided a better understanding of the relationship between blood tests and the malignancies analyzed. Notably, nodding (branching) across malignancies grouped profiles with low correlation values, such as C15 esophagus and C19 rectum. Furthermore, it aggregated C16 stomach and C18 colon, which substantially correlated with particular blood tests, such as RDW for C16 stomach and erythrocytes for C18 colon. Nevertheless, an extra node distinguished C16 stomach from the others (also influenced by hemoglobin and hematocrit, in accordance with PCA). Remarkably, these findings are coherent with recent studies that relate hematological parameters as predictors of diagnosis and prognosis of digestive tract malignancies (21). Moreover, Pietrzyk et al. found that RDW alone could discriminate patients with gastric cancer from healthy individuals (22). Yazici et al. described RDW as a prognostic gastric cancer biomarker with elevated values associated with short-term mortality (23). In colorectal carcinoma, Kinar et al. used hemoglobin, hematocrit, RDW, MCH, MCHC, and MCV to diagnose, with sensitivity stability between 480 and 240 days before diagnosis (AUC 0.81 in an external evaluation set of 5,000+ patients) (12).

The ability to associate routine blood tests to distinguish digestive tract tumors at the time of diagnosis was herein evaluated through the computation of univariate and multivariate analysis. While the univariate analysis confirmed the feature importance described in the heat map, the multivariate analysis computed on a binomial LR with the four predictors enhanced the discriminatory ability for each tumor. C18 colon had the most significant AUC of 0.77, with 94% sensitivity and 52% specificity, followed by C16 stomach, which had an AUC of 0.68, with 52% sensitivity and 80% specificity. C15 esophagus and C19 rectum were less predictable, with an AUC of 0.58 and 0.48, respectively. Interestingly, although C18 colon and C19 rectum were similarly patterned (PCA), their discrimination from the others was considerably different, demonstrating that comparable patterns are not accurately anticipated until feature correlation maintains higher correlation values (heat map and ROC analysis).

This study presents some limitations, which we next highlight: the small sample size in each tumor category (particularly in C15 esophagus and C19 rectum) and the research's retrospective, single-center nature. Yet, because this strategy focused on recognizing disease profiles, blood tests from the ICD-10 codification date were valued more. Nonetheless, the given performance metrics are virtuous and promising, especially because comparisons were made between diseased patients only (without healthy volunteers) and predicted using routine blood parameters, which implies an additional potential of this methodological approach. We believe that the novelty generated by this study will trigger further multicentric studies to further validate the current findings. Furthermore, the ability to evaluate profile changes over time and correlate them with labeled stages of the disease may enable the development of a point-of-care follow-up map, extracting additional value from routine blood assessments.

## Conclusion

Patterns of prevalent digestive tract tumors were recognized and categorized, considering the hematological results of the hemoglobin concentration, and the RDW, erythrocytes, and hematocrit counts from the date of ICD-10 codification. Tumor profiles were decomposed in agreement with the anatomical location of tumor development, separating the C18 colon and C19 rectum from the C15 esophagus and those from the C16 stomach, confirming the ability of hematological parameters to perform profile distinction in digestive tract tumors.

Both the heat map analysis and the multivariate binary logistic regression confirmed the importance of higher erythrocyte count in distinguishing C18 colon from other malignancies (AUC = 0.77, 94% sensitivity, and 52% specificity) and the importance of both a high number of RDW with low levels of hemoglobin, hematocrit, and erythrocytes in distinguishing C16 stomach from the remaining tumors (AUC = 0.68, 52% sensitivity, 80% specificity). Although C15 esophagus and C19 rectum were less predicted, this study demonstrates that routine blood tests have the potential predictive capacity to distinguish digestive tract tumors at diagnosis.

## Data availability statement

The raw data supporting the conclusions of this article will be made available by the authors, without undue reservation.

## Ethics statement

The studies involving humans were approved by Braga Hospital Ethics Committee. The studies were conducted in accordance with the local legislation and institutional requirements. Written informed consent for participation was not required from the participants or the participants' legal guardians/next of kin in accordance with the national legislation and institutional requirements.

## Author contributions

MS-S, MMaj, and NS contributed to the conception and design of the study. MMac contributed to data extraction with JR and MMaj support in coordination and validation. MS-S, NS, and JS contributed to data analysis and interpretation. MS-S drafted the manuscript with NS and JS feedback, proofreading, and content curation. All authors approved the manuscript. All authors guaranteed the integrity of the study.

## References

[1] Torre LA Bray F Siegel RL Ferlay J Lortet-Tieulent J Jemal A. Global cancer statistics, 2012. CA Cancer J Clin. (2015) 65:87–108. 10.3322/caac.21262 25651787

[2] Bray F Ferlay J Soerjomataram I Siegel RL Torre LA Jemal A. Global cancer statistics 2018: GLOBOCAN estimates of incidence and mortality worldwide for 36 cancers in 185 countries. CA Cancer J Clin. (2018) 68:394–424. 10.3322/caac.21492 30207593

[3] Pasechnikov V Chukov S Fedorov E Kikuste I Leja M. Gastric cancer: prevention, screening and early diagnosis. World J Gastroenterol. (2014) 20:13842–62. 10.3748/wjg.v20.i38.13842 25320521 PMC4194567

[4] Kanth P Inadomi JM. Screening and prevention of colorectal cancer. BMJ. (2021) 374:n1855. 10.1136/bmj.n1855 34526356

[5] Necula L Matei L Dragu D Neagu AI Mambet C Nedeianu S. Recent advances in gastric cancer early diagnosis. World J Gastroenterol. (2019) 25:2029–44. 10.3748/wjg.v25.i17.2029 31114131 PMC6506585

[6] Hallworth MJ. The ‘70% claim': what is the evidence base? Ann Clin Biochem. (2011) 48:487–8. 10.1258/acb.2011.011177 22045648

[7] Matthew R Pincus NZ. AJ interpreting laboratory results. In:Henry JB McPherson RA Pincus MR, editors. Henry's Clinical Diagnosis and Management 22th Edition.pdf. Philadelphia, PA: Elsevier/Saunders (2011). p. 92–4.

[8] Spell DW Jones DV Harper WF Bessman JD. The value of a complete blood count in predicting cancer of the colon. Cancer Detect Prev. (2004) 28:37–42. 10.1016/j.cdp.2003.10.002 15041076

[9] Han F Liu Y Cheng S Sun Z Sheng C Sun X. Diagnosis and survival values of neutrophil-lymphocyte ratio (NLR) and red blood cell distribution width (RDW) in esophageal cancer. Clin Chim Acta. (2019) 488:150–8. 10.1016/j.cca.2018.10.042 30389457

[10] Wei TT Wang LL Yin JR Liu YT Qin BD Li JY. Relationship between red blood cell distribution width, bilirubin, and clinical characteristics of patients with gastric cancer. Int J Lab Hematol. (2017) 39:497–501. 10.1111/ijlh.12675 28497572

[11] Goldshtein I Neeman U Chodick G Shalev V. Variations in hemoglobin before colorectal cancer diagnosis. Eur J Cancer Prev. (2010) 19:342–4. 10.1097/CEJ.0b013e32833c1be0 20543703

[12] Kinar Y Kalkstein N Akiva P Levin B Half EE Goldshtein I. Development and validation of a predictive model for detection of colorectal cancer in primary care by analysis of complete blood counts: a binational retrospective study. J Am Med Informatics Assoc. (2016) 23:879–90. 10.1093/jamia/ocv195 26911814 PMC4997037

[13] Hornbrook MC Goshen R Choman E O'Keeffe-Rosetti M Kinar Y Liles EG. Early colorectal cancer detected by machine learning model using gender, age, and complete blood count data. Dig Dis Sci. (2017) 62:2719–27. 10.1007/s10620-017-4722-8 28836087

[14] Soerensen PD Christensen H Gray Worsoe Laursen S Hardahl C Brandslund I Madsen JS. Using artificial intelligence in a primary care setting to identify patients at risk for cancer: a risk prediction model based on routine laboratory tests. Clin Chem Lab Med. (2022) 60:2005–16. 10.1515/cclm-2021-1015 34714986

[15] Mahmood N Shahid S Bakhshi T Riaz S Ghufran H Yaqoob M. Identification of significant risks in pediatric acute lymphoblastic leukemia (ALL) through machine learning (ML) approach. Med Biol Eng Comput. (2020) 58:2631–40. 10.1007/s11517-020-02245-2 32840766

[16] Meiseles A Paley D Ziv M Hadid Y Rokach L Tadmor T. Explainable machine learning for chronic lymphocytic leukemia treatment prediction using only inexpensive tests. Comput Biol Med. (2022) 145:105490. 10.1016/j.compbiomed.2022.105490 35405402

[17] Sarker IH. Machine learning: algorithms, real-world applications and research directions. SN Comput Sci. (2021) 2:160. 10.1007/s42979-021-00592-x 33778771 PMC7983091

[18] Bruckert S Finzel B Schmid U. The next generation of medical decision support: a roadmap toward transparent expert companions. Front Artif Intell. (2020) 3:507973. 10.3389/frai.2020.507973 33733193 PMC7861251

[19] Gunčar G Kukar M Notar M Brvar M Cernelč P. Application of machine learning for hematological diagnosis. Sci Rep. (2018) 8:411. 10.1038/s41598-017-18564-8 29323142 PMC5765139

[20] Tang S Wu WK Li X Wong SH Wong N Chan MT. Stratification of digestive cancers with different pathological features and survival outcomes by microRNA expression. Sci Rep. (2016) 6:1–10. 10.1038/srep24466 27080237 PMC4832245

[21] Jung DH Lee HJ Han DS Suh YS Kong SH Lee KU. Impact of perioperative hemoglobin levels on postoperative outcomes in gastric cancer surgery. Gastric Cancer. (2013) 16:377–82. 10.1007/s10120-012-0196-8 23007652

[22] Pietrzyk L Plewa Z Denisow-Pietrzyk M Zebrowski R Torres K. Diagnostic power of blood parameters as screening markers in gastric cancer patients. Asian Pacific J Cancer Prev. (2016) 17:4433–7. 10.7314/APJCP.2016.17.9.4433 27797257

[23] Yazici P Demir U Bozkurt E Isil GR Mihmanli M. The role of red cell distribution width in the prognosis of patients with gastric cancer. Cancer Biomark. (2017) 18:19–25. 10.3233/CBM-160668 27814271 PMC13020618

